# Supplementary material for: Antibacterial Activity Potential of Industrial Food Production Waste Extracts against Pathogenic Bacteria: Comparative Analysis and Characterization
Source: Foods. 2024 Jun 17;13(12):1902. doi: 10.3390/foods13121902 (PMC11203380; doi:10.3390/foods13121902)
Supplement: Supplementary file 1 [file foods-13-01902-s001.zip › foods-3032421-supplementary.pdf]

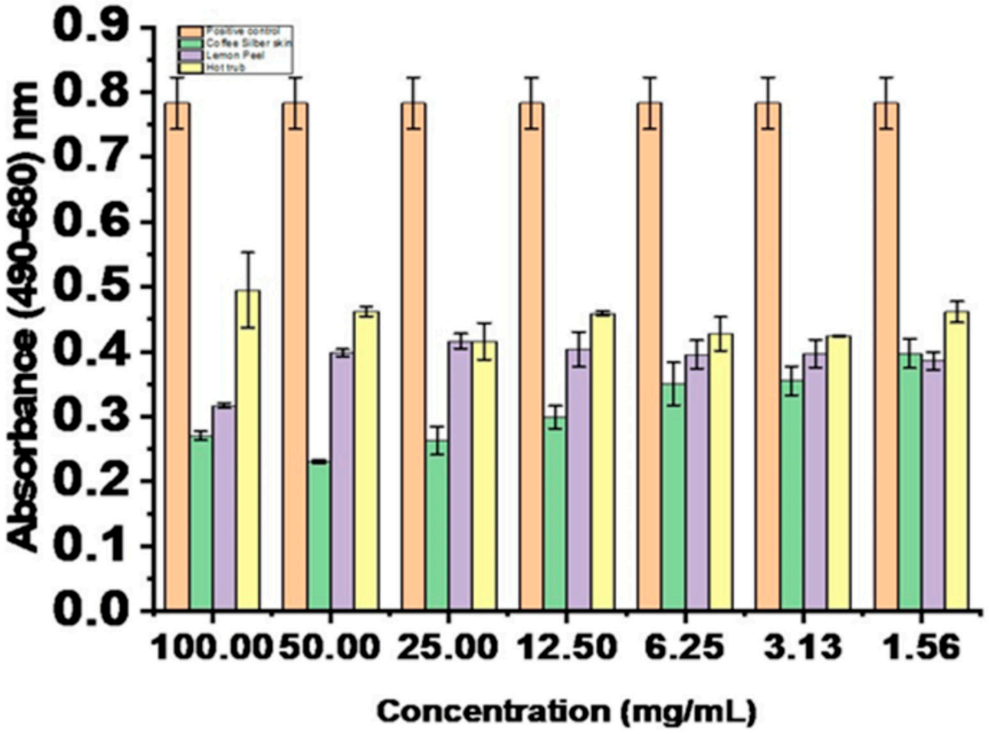

Figure S1. Cytotoxicity effect of Coffee Silverskin, Hot trub, and Lemon peel phytoextracts against HaCaT cells.
